# Low revision rate of dual mobility cups after arthroplasty for acute hip fractures: report of 11,857 hip fractures in the Dutch Arthroplasty Register (2007–2019)

**DOI:** 10.1080/17453674.2020.1845031

**Published:** 2020-11-11

**Authors:** Esther M Bloemheuvel, Liza N Van Steenbergen, Bart A Swierstra

**Affiliations:** a Department of Orthopedic Surgery, Sint Maartenskliniek , Nijmegen ;; b Dutch Arthroplasty Register (LROI), ’s Hertogenbosch , the Netherlands

## Abstract

Background and purpose — Dislocation is one of the most frequent reasons for cup revision after total hip arthroplasty (THA) for an acute fracture. A dual mobility cup (DMC) might reduce this risk. We determined the cup revision rate after THA for an acute fracture according to type of cup.

Patients and methods — All THAs for an acute fracture registered in the Dutch Arthroplasty Register (LROI) during 2007–2019 were included (n = 11,857). Type of cup was divided into DMC and unipolar cup (UC). Competing risk analyses were performed with cup revision for any reason as endpoint. Multivariable Cox regression analyses with outcome cup revision were performed adjusted for sex, age, ASA class, and surgical approach, stratified for UC THA with femoral head size of 32 mm and 22–28 mm.

Results — A DMC was used in 1,122 (9%) hips. The overall 5-year cup revision rate for any reason after THA for acute fracture was 1.9% (95% CI 1.6–2.2). Cup revision for dislocation within 5 years was performed in 1 of 6 DMC THAs versus 108 of 185 (58%) UC THAs. Univariable Cox regression analyses showed no statistically significant difference in cup revision rate between DMC and UC (HR = 0.8; CI 0.4–1.5). Multivariable Cox regression analyses showed lower risk of cup revision in DMC THA (n = 1,122) compared with UC THA with 22–28 mm femoral head size (n = 2,727) (HR = 0.4; CI 0.2–0.8).

Interpretation — The 5-year cup cumulative incidence of revision after THA for acute fracture was comparable for DMC and UC THA. However, DMC THA had a lower risk of cup revision than UC THA with 22–28 mm femoral head.

The risk for revision in case of total hip arthroplasty (THA) after an acute fracture is higher than after hemiarthroplasty (Parker et al. 2010). Dislocation is one of the most frequent reasons for cup revision after an acute fracture (Gjertsen et al. 2007). We have shown low cup revision rates for dislocation using dual mobility cup (DMC) THA in patients with osteoarthritis (Bloemheuvel et al. 2019). The use of DMC in THA after an acute fracture might therefore be beneficial to prevent this complication. At the same time also femoral head size (in unipolar cups [UC]) and surgical approach influence the risk of revision for dislocation (Byström et al. 2003, Hailer et al. 2012, Kostensalo et al. 2013, Zijlstra et al. 2017).

We hypothesized that the cup revision rate for dislocation in THA for acute fracture is lower with DMC than UC but that this can be affected by femoral head size (in UC) and surgical approach. We therefore determined the cup revision rate because of dislocation after THA for an acute fracture according to type of cup and head size.

## Patients and methods

The Dutch Arthroplasty Register (LROI) started in 2007 and has a completeness of 98% for primary and revision hip arthroplasty (www.lroi-report.nl). The LROI database contains patient, procedure, and prosthesis characteristics. For each component a product number is registered to identify the characteristics of the prosthesis, such as dual mobility or unipolar cup.

The vital status of all patients is obtained actively on a regular basis from Vektis, the national insurance database on health care in the Netherlands, which records all deaths of Dutch citizens (www.vektis.nl).

For this study we included all primary THAs in the period 2007–2019 with a diagnosis of acute fracture. A cup revision was defined as a procedure where at least the cup or the cup and liner were exchanged or removed. Closed reduction after a dislocation or incision and drainage for infection without component exchange are not included in the LROI.

**Figure 1.**
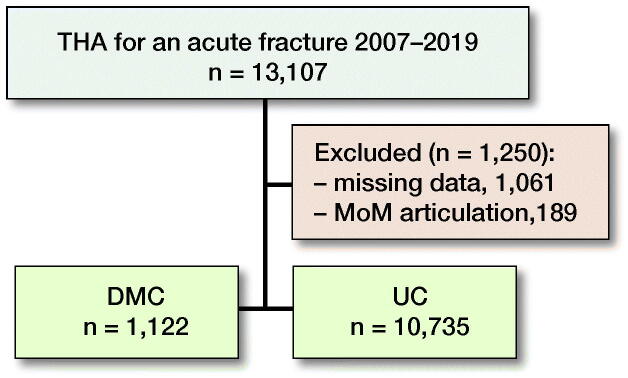
Patient flow.

Records with a missing cup product number (n = 1,061) and metal-on-metal hip arthroplasties were excluded (n = 189). 11,857 primary THAs were included and divided into 2 groups: DMC THA and UC THA (Figure 1).

### Statistics

UC THA and DMC THA were described separately concerning patient and procedure characteristics. Survival time was calculated as the time from primary THA to cup revision for any reason, death of the patient, or end of follow-up (December 31, 2019). Cumulative crude incidence of cup revision was calculated using competing risk analysis, where death was considered to be a competing risk (Lacny et al. 2015, Wongworawat et al. 2015).

Multivariable Cox regression analyses were performed to compare DMC and UC THA. Adjustments were made for sex, age, ASA class, and surgical approach and stratified by UC femoral head size (22–28 mm and 32 mm). BMI and smoking status were not included as covariates, since these have only been available in the LROI database since 2014.

For all covariates added to the model, the proportional hazards assumption was checked by inspecting log-minus-log curves and met.

Reasons for cup revision were described and compared using a chi-square test. P-values below 0.05 were considered statistically significant. For the 95% confidence intervals (CI), we assumed that the number of observed cases followed a Poisson distribution.

### Ethics, data sharing, funding, and potential conflicts of interests

The LROI uses the opt-out system to require the informed consent of patients. The dataset was processed in compliance with the regulations of the LROI governing research on registry data. Data are available from the LROI but restrictions apply to the availability of these data, which were used under license for the current study. No external funding was received. No competing interests were declared.

## Results

11,857 THAs for acute fracture were included. In 9% a DMC THA and in 91% a UC THA was used. The median follow-up was 3.4 years (0–13), with 35% of records having a follow-up period of 5 years or longer.

Of all included acute fracture THA patients, 26% (CI 22–31) in the DMC THA group died and 16% (CI 15–17) in the UC THA groups died within 5 years of the primary procedure.

**Figure 2.**
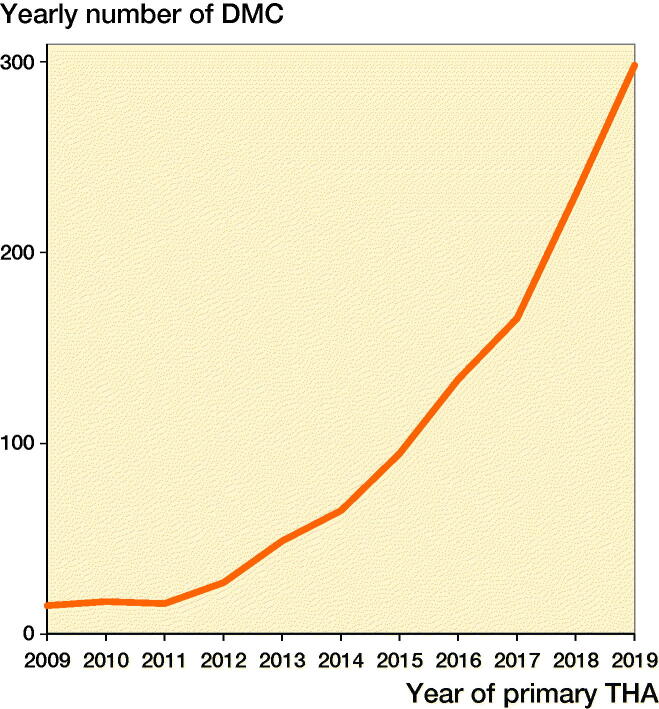
Use of DMC THA in case of an acute fracture in the period 2009–2019 in the Netherlands.

**Figure 3.**
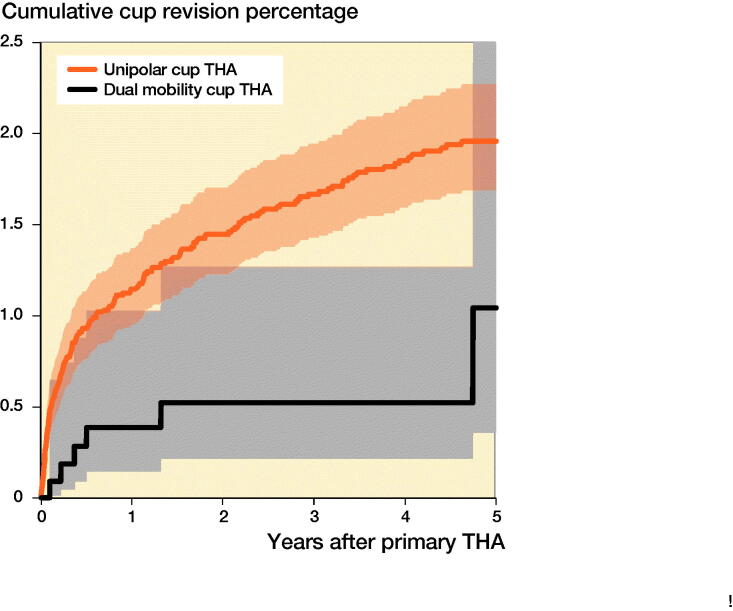
Crude cumulative overall cup revision rate of THAs for acute fracture according to type of cup.

The use of a DMC THA in acute fracture patients increased from 15 in 2009 (3% of all THAs) to 299 (18% of all THAs) in 2019 (Figure 2). The mean age was 70 years in both groups. The proportion ASA class III–IV was higher in the DMC THA group (40%) compared with the UC DMC group (24%). In 70% the DMC THA was cemented compared with 32% in the UC THA group. The most frequent approach was posterolateral in both groups (Table 1). In the UC THA group, most often a 32 mm head was used (51%). There were 2,727 (26%) small-sized heads used (22–28 mm) and 23% had a 36 mm head size. The overall 5-year cumulative incidence of cup revision rate for any reason after THA for acute fracture was 1.9% (CI 1.6–2.2) with 6 of 1,122 cup revisions for DMC THA and 185 of 10,735 cup revisions for UC THA. The 5-year cumulative incidence of cup revision rate for DMC THA was 1.0% (CI 0.4–3.0) and 2.0% (CI 1.7–2.3) for UC THA (Figure 3). In UC THA with 36 mm heads the 5-year cumulative incidence of cup revision rate was 1.4% (CI 0.9–2.0) and for UC THA with 32 mm heads this was 1.7% (CI 1.3–2.1), while for UC THA with 22–28 mm heads this was 2.7% (CI 2.2–3.4).

**Table 1.** Patient characteristics of THAs for acute fracture according to type of cup (n = 11,857). Values are count (%) unless otherwise specified

	DMC THA	UC THA
Factor	n = 1,122	n = 10,735
Sex		
Male	382 (34)	3,324 (31)
Female	738 (66)	7,395 (69)
Age, median (p5–p95) **^a^**	70 (52–86)	70 (54–84)
Previous operation on affected hip		
Yes	108 (10)	650 (6)
No	1,002 (90)	9,749 (94)
ASA score		
I	75 (7)	1,702 (16)
II	595 (53)	6,153 (59)
III–IV	448 (40)	2,531 (25)
Fixation		
Cemented	776 (70)	3,344 (32)
Hybrid (acetabulum cemented)	135 (12)	327 (3)
Hybrid (femur cemented)	58 (5)	997 (9)
Cementless	137 (13)	5,923 (56)
Approach		
Anterior	61 (5)	1,142 (11)
Anterolateral	10 (1)	874 (8)
Direct lateral	84 (8)	2,345 (22)
Posterolateral	955 (85)	6,240 (59)
Other	8 (1)	34 (0)
Head diameter, mm		
22–28	1,094 (100)	2,727 (26)
32	3 (0)	5,380 (51)
36		2,382 (23)
≥ 38		60 (0)

**^a^** 5th percentile to 95th percentile

DMC: dual mobility cup; UC: unipolar cup.

Numbers do not add up to total due to missing data.

Univariable as well as multivariable Cox regression analyses showed a statistically significant lower risk for cup revision in the DMC THA group compared with UC THA with a 22–28 mm femoral head (HRadjusted 0.4 [0.2–0.8]), but no statistically significant difference in cup revision rate between DMC and UC THA with a 32 mm femoral head (HRadjusted 0.6 [CI 0.3–1.2]) (Table 2).

**Table 2.** Multivariable Cox regression analysis for DMC THA and UC THA compared with UC THA 32 mm femoral head and 22–28 mm femoral head in acute fracture patients

Type of hip prosthesis	n	crude HR	adjusted HR **^a^**
DMC THA	1,122	0.7 (0.4–1.4)	0.6 (0.3–1.2)
UC THA 32 mm head	5,380	1.0 (ref.)	1.0 (ref.)
DMC THA	1,122	0.5 (0.2–0.9)	0.4 (0.2–0.8)
UC THA 22–28 mm head	2,727	1.0 (ref.)	1.0 (ref.)

**^a^** Adjusted for age, sex, ASA classification, and surgical approach.

1 of 6 DMC THAs were revised for dislocation versus 108 of 185 (58%) UC THAs. (Suspicion of) infection (3/6) and cup loosening (4/6) were other registered reasons for cup revision in the DMC group, compared with 23/185 (12%) and 29/185 (16%) in the UC group (Table 3).

**Table 3.** Reason for cup revision within 5 years according to type of cup

	DMC THA	UC THA
Factor	n = 1,122	n = 10,735
All cup revisions within 5 years	6	185
Reason for revision ^a^		
Dislocation	1	108
Infection	3	23
Wear	0	5
Periprosthetic fracture	0	13
Loosening femoral component	1	18
Loosening acetabular component	4	29
Peri-articular ossification	1	2
Other	2	32

DMC: Dual mobility cup; UC: Unipolar cup.

**^a^** The sum is higher than the total amount since more than 1 reason for revision can be registered.

## Discussion

We found that DMC is increasingly used in THA for acute fractures. The clinicians’ expectation to reduce the risk for dislocation is the most probable reason to use this more expensive cup. We found 6 cup revisions within 5 years when a DMC THA was used, and only 1 of these 6 was revised for dislocation. Our focus on short-term revision rates is justified as the majority of dislocations occur early after the index operation (Enocson et al. 2009).

In the Nordic Arthroplasty Register Association (NARA) a reduced revision risk for DMC in THA for acute femoral neck fracture has been shown by Jobory et al. (2019). They matched 4,520 hip fractures treated with a DMC THA to 4,520 hip fractures with UC THA and found a lower risk for cup revision for dislocation for DMC, with a hazard ratio of 0.32 adjusted for approach. However, they only included head size 32 and 36 mm in contrast to our study in which head sizes 22–28 mm were included as well. Tabori-Jensen et al. (2019) found low dislocation rates of DMC THA after acute femoral neck fracture in a cohort study of 966 hips. After mean 5.4 years follow-up, 8 cups were revised, 3 due to repeated dislocations. Their findings are comparable to our results.

We found a statistically significant lower risk for cup revision in the DMC THA group compared with UC THA with a 22–28 mm femoral head. This is in accordance with our hypothesis and with the findings of Kostensalo et al. (2013), based on data from the Finnish Arthroplasty Register, who found a reduced dislocation revision rate in head sizes > 28 mm. Comparable results were found in register studies from Norway (Byström et al. 2003), Sweden (Hailer et al. 2012) and the Netherlands (Zijlstra et al. 2017).

Our hypothesis that surgical approach might influence the (cup) revision rate could not be confirmed. This influence has been shown in another recent LROI study by Moerman et al. (2018), who found that posterolateral approach was a risk factor compared with other approaches (HR 1.0 versus 0.7) for revision in case of THA or hemiarthroplasty for hip fracture (74% of their study population underwent a hemiarthroplasty). Also based on LROI data, Zijlstra et al. (2017) showed that the posterolateral approach resulted in higher revision rates due to dislocation compared with all other surgical approaches (HR = 1.0 vs. 0.5–0.6) in the case of THA for primary osteoarthritis.

A strength of our study is the focus on cup revisions only, since type of revision (cup, stem, insert, and/or femoral head exchange) is specified in the LROI.

A limitation of register studies is the risk for selection bias. First, there is a possibility that DMC was used exclusively in a few clinics and/or by single surgeons because of preference. Second, it is possible that different cup designs were used for different types of patients for other reasons such as patient comorbidity. We tried to make an estimation of frailty and comorbidity using patient characteristics available in the LROI and found no statistically significant differences between the 2 groups based on age and ASA classification. We plan further analyses with a more extensive set of patient variables including smoking status, Charnley score and BMI.

Another limitation of this study is the fact that an acute hip fracture was not further specified in the LROI database. Most often an acute femoral neck fracture will have been the indication for a THA, but some trochanteric fractures cannot be ruled out.

Closed reductions for dislocations are not registered in the LROI. Reductions for UC THA can often be performed without surgery, but closed reductions are often impossible in DMC THA needing surgery with component exchange and hence registration in the LROI. This means that the dislocation revisions in DMC reflect the number of postoperative dislocations better than the dislocation revisions in UC.

In conclusion, the 5-year cumulative incidence of cup revision rate after THA for acute fracture was 1.9% (CI 1.6–2.2) being comparable for DMC and UC THA with a 32 mm femoral head. However, DMC THA had a lower risk of cup revision than UC THA with a 22–28 mm femoral head.
